# Real-World Outcomes of Stage IV NSCLC with PD-L1 ≥ 50% Treated with First-Line Pembrolizumab: Uptake of Second-Line Systemic Therapy

**DOI:** 10.3390/curroncol30060402

**Published:** 2023-05-26

**Authors:** Rebekah Rittberg, Bonnie Leung, Aria Shokoohi, Alexandra Pender, Selina Wong, Zamzam Al-Hashami, Ying Wang, Cheryl Ho

**Affiliations:** 1 CancerCare Manitoba, Winnipeg, MB R3E 0V9, Canada; 2 BC Cancer Vancouver, Vancouver, BC V5Z 4E6, Canada; 3 Faculty of Medicine and Dentistry, University of Alberta, Edmonton, AB T6G 2R7, Canada; 4 The Institute of Cancer Research, London SW7 3RP, UK; 5 BC Cancer Victoria, Victoria, BC V8R 6V5, Canada; 6 Sultan Qaboos Comprehensive Cancer Care and Research Center, Muscat 123, Oman

**Keywords:** real-world evidence, PDL1, pembrolizumab, NSCLC, second line

## Abstract

Introduction: Platinum-based chemotherapy was compared to single-agent pembrolizumab in advanced non-small cell lung cancer (NSCLC) with PDL1 > 50% in KEYNOTE-024. In this trial, it was found that patients who received single-agent pembrolizumab had improved progression-free survival in addition to overall survival (OS). Based on KEYNOTE-024, only 53% of patients treated originally with pembrolizumab received second-line anticancer systemic therapy with an OS of 26.3 months. Based on these results, the objective of this study was to characterize real-world NSCLC patients who received second-line therapy after single-agent pembrolizumab. Methods: This was a retrospective cohort study considering stage IV NSCLC patients diagnosed with BC Cancer between 2018 and 2021 with PD-L1 ≥ 50% who received first-line single agent pembrolizumab. Patient demographics, cancer history, treatment administered, and survival were collected retrospectively. Descriptive statistics were produced. OS was calculated using Kaplan–Meier curves and compared using the log rank test. A multivariate model evaluated characteristics associated with the receipt of second-line therapy. Results: A total of 718 patients were diagnosed with Stage IV NSCLC and received at least one cycle of pembrolizumab. The median duration of treatment was 4.4 months, and the follow-up duration was 16.0 months. There were 567 (79%) patients who had disease progression, of whom 21% received second-line systemic therapy. Within the subset of patients with disease progression, the median duration of treatment was 3.0 months. It would be found that patients who received second-line therapy had better baseline ECOG performance status, were younger at diagnosis, and had a longer duration of pembrolizumab. Within the full population, the OS from the treatment initiation date was 14.0 months. OS was 5.6 months in patients who did not receive additional therapy after progression and 22.2 months in patients who received subsequent therapy. Baseline ECOG performance status was associated with improved OS in multivariate analysis. Conclusion: Based on this real-world Canadian population, 21% of patients received second-line systemic therapy, despite second-line therapy being associated with prolonged survival. In this real-world population, we found that 60% fewer patients received second-line systemic therapy when compared to KEYNOTE-024. Although differences always exist when comparing a clinical and non-clinical trial population, our findings suggest undertreating stage IV NSCLC patients.

## 1. Introduction

Lung cancer continues to be the global leader in cancer-related death, with parallel rates of incidence and mortality due to the high rate of new diagnosis in advanced stages and the high rate of disease recurrence even in early stages [1,2]. In 2018 alone, there were a total of 2.1 million new lung cancer cases and 1.8 million deaths due to lung cancer [1]. Non-small cell lung cancer (NSCLC) accounts for approximately 80–85% of all new lung cancer diagnoses, with dramatic improvements in treatment options and outcomes over the last 15 years [3]. First-line platinum-doublet chemotherapy had been the standard of care until 2005; however, since then, there has been the addition of maintenance pemetrexed in patients with non-squamous histology, the identification and targeting of driver mutations, and immunotherapy [4,5,6].

First-line systemic therapy in patients without an identified driver mutation is dependent on programmed cell death ligand 1 (PD-L1) status. Patients with PD-L1 ≥ 50% can be treated with a single-agent immune checkpoint inhibitor (ICI), which was found to have superior outcomes compared to platinum doublet chemotherapy in addition to being more tolerable for both squamous and non-squamous histology [6]. Pembrolizumab, a programmed cell death 1 (PD-1) monoclonal antibody, was found to have improved progression-free survival (PFS) and overall survival (OS) compared to platinum doublet chemotherapy in KEYNOTE-024 [7,8]. IMpower110 similarly found that atezolizumab, a PD-L1 monoclonal antibody, compared to platinum doublet improved PFS and OS [9]. EMPOWER-Lung 1 also found improved PFS and OS with cemiplimab monotherapy compared to platinum doublet [10].

Other options for first-line treatment in the PDL1 ≥ 50% population include platinum doublet alone for the immunotherapy-ineligible population and combination platinum-based chemotherapy with ICI. A pooled retrospective, exploratory analysis of registration trials including ICI alone, chemotherapy alone, and chemotherapy plus ICI was conducted by the Food and Drug Administration (FDA). This hypothesis-generating study in patients with PDL1 ≥ 50% did not suggest a difference in OS outcomes, and older adults ≥ 75 may have better OS outcomes with the immunotherapy alone option [11].

Due to the results of the phase 3 trials, the supporting evidence from the FDA analysis, and excellent tolerability, single agent ICI is now widely accepted as a first-line systemic therapy option for patients with stage IV NSCLC non-squamous and squamous histology harboring a PD-L1 ≥ 50% with clinical trial median OS ranging from 20.2 to 26.3 months [7,9,10].

Inevitably, disease progression occurs, and second-line systemic therapy is generally platinum doublet chemotherapy followed by third-line docetaxel [6]. Significant attrition occurs in the receipt of second- and third-line systemic therapy in patients with stage IV NSCLC, with just 53% of the clinical trial population in KEYNOTE-024 receiving second-line or later therapy [8]. When considering real-world outcomes, there is an even lower rate of second-line systemic therapy uptake, with significant variation depending on first-line therapy selection [12].

In this real-world evaluation of Stage IV NSCLC with PD-L1 ≥ 50% treated with single agent first-line pembrolizumab, we evaluate the rate of second-line therapy after pembrolizumab and predictive factors of second-line therapy in addition to evaluating patient outcomes.

## 2. Methods

### 2.1. Population

British Columbia is a Canadian province with a population of 5.1 million people. Oncology treatment is centralized through BC Cancer, which includes six cancer centers and over 40 satellite community oncology network sites. Baseline characteristics, diagnostic information, and survival outcomes for all referred lung cancer patients in British Columbia, Canada, are maintained in the outcomes and surveillance integration system, allowing for a provincial analysis.

A retrospective cohort study was conducted at BC Cancer, evaluating patients diagnosed with Stage IV NSCLC with PD-L1 ≥ 50% treated with first-line pembrolizumab between 1 January 2018 and December 31, 2021. The date of censoring was 24 May 2022. Baseline patient demographics, eastern cooperative oncology group (ECOG) performance status, cancer staging, treatment, and survival were collected using the outcomes and surveillance integration system, electronic medical records, and the billing administration database for chemotherapy. Baseline demographic information included patient age at the time of diagnosis, sex (female or male), histology (squamous or non-squamous), smoking status (never, former, active, or unknown smoking status), smoking pack years, and baseline mutation status. Radiographic imaging, including central nervous system (CNS) imaging, was conducted at the physician’s discretion at baseline and during treatment.

First-line pembrolizumab was administered every three weeks up to a total of 35 cycles or every six weeks up to 17 cycles. When administered without delay, this equals approximately two years of treatment [7,8]. Second-line therapy was defined as any systemic therapy, including chemotherapy or targeted therapy, delivered after first-line pembrolizumab. The treatment duration was defined as the time from the first treatment administration to the last treatment administration.

PD-L1 status was confirmed using 22C3 PharmDx PD-L1 assays and reported clinically as the percent of tumor cells with membrane staining. Molecular characterization by the next-generation sequencing (NGS) panel was funded for non-squamous, mixed, and selected squamous patients. Data on select driver mutations were collected, including ALK, BRAF, EGFR, KRAS, MET, and ROS1.

### 2.2. Statistical Analysis

Descriptive statistics were produced evaluating patient and oncologic characteristics using Chi-square and Mann–Whitney U tests. OS was calculated from the date of treatment initiation using Kaplan–Meier curves and compared using the log rank test. The Cox proportional hazards model was used for multivariate analysis of the impact of independent variables on OS. A comparison of OS was made between the patients who received the best supportive care and those who received second-line treatment using the date of the last pembrolizumab infusion as the baseline date, acting as a proxy for progression. Patients were censored at the time of the last known follow-up. A logistic regression multivariate model evaluated characteristics associated with the receipt of second-line systemic therapy. The statistically significant *p*-value was <0.05. The statistical package for the social sciences, version 28, was used for all analyses.

### 2.3. Ethics Statement

This study was conducted with the University of British Columbia/BC Cancer Research Ethics Board approval (H18-037444). A waiver of consent was granted to extract and analyze data for this retrospective review.

## 3. Results

Between 2018 and 2021, a total of 718 patients with stage IV NSCLC received first-line pembrolizumab at BC Cancer. Baseline characteristics are found in Table 1. The median patient age at the time of diagnosis was 70 years, with 80% of the population having non-squamous histology. ECOG performance status 0–1 made up 61% of the population, with the remainder of the population having a performance status of ≥2. The majority of the population had a smoking history, with 73% of patients being past smokers and 18% of the population being current smokers. CNS metastases were known at baseline in 9% of the population. KRAS mutations were identified in 32% of the non-squamous histology population, with G12C being the most common (54%).

**Figure 1:**
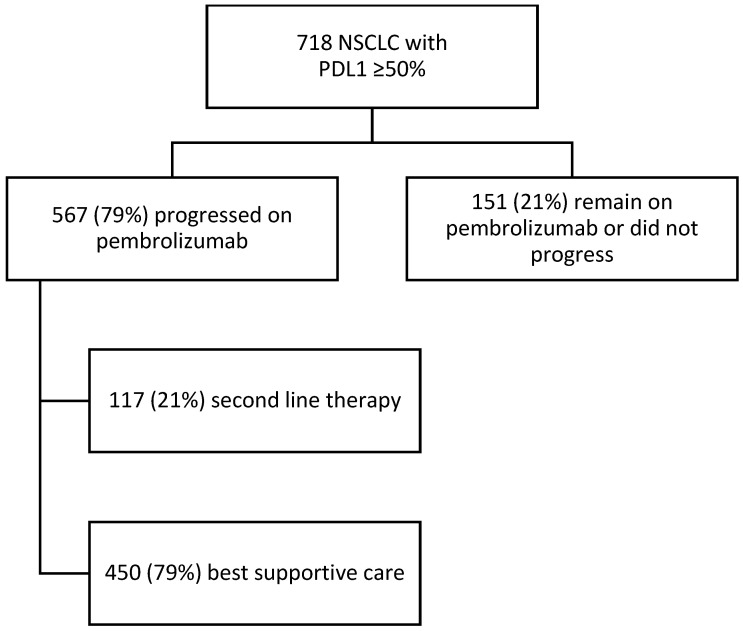
Consort diagram of NSCLC with PDL1 ≥ 50% population treated with pembrolizumab.

The median follow-up within the population was 16.0 months, with a median duration on first-line pembrolizumab of 4.4 months. At the time of data cutoff, 21% (*n* = 151) of patients had not experienced radiographic evidence of disease progression, with 10% (*n* = 72) patients completing 2 years/35 cycles of pembrolizumab. Disease progression was observed in 567 patients, of whom 21% (*n* = 117) received second-line systemic therapy (Figure 1). Within this subset of patients who experienced disease progression, the median duration of pembrolizumab treatment was 3.0 months, with 195 (34%) of therapy within 9 weeks. The follow-up duration was 11.7 months.

Patients who received second-line therapy were younger and had a better baseline ECOG performance status (Table 1). Within the patients with baseline ECOG performance status of 0–1, 21% received at least two lines of therapy, compared to patients with baseline ECOG performance status of ≥2, with just 10% receiving at least two lines of therapy. There was no difference in sex (*p* = 0.519), smoking status (*p* = 0.416), or histology (*p* = 0.136). Patients who received second-line therapy had a longer duration of treatment with pembrolizumab compared to patients who received the best supportive care (5.4 months compared to 2.6 months). In the multivariate model including age, ECOG performance status, and duration on pembrolizumab, each independently impacted receipt of second-line therapy (Table 2). Second-line systemic therapy was carboplatin doublet for 103 patients (88%), other chemotherapy for 14 patients (12%), and tyrosine kinase inhibitors for 7 patients (6%).

**Figure 2:**
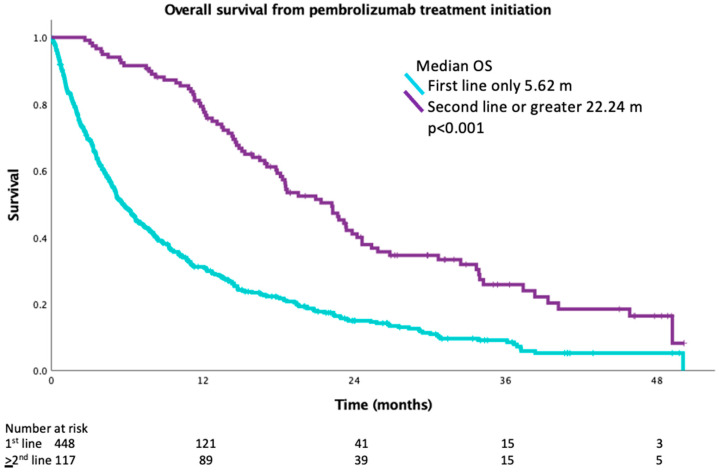
OS of patients from treatment initiation who received pembrolizumab only versus second line or greater therapy.

**Figure 3:**
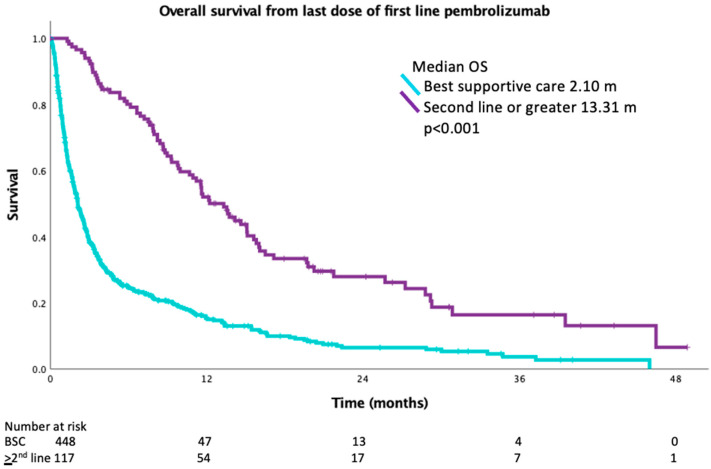
Comparison of OS of patients who had progression on first line pembrolizumab from time of last dose of pembrolizumab who received best supportive care versus second line or greater therapy.

The median OS from the date of treatment initiation was 14.0 months (95% CI 11.8–16.2) in the entire population. Median OS was 5.6 months (95% CI 4.8–6.5) in patients who did not receive additional therapy after progression and 22.2 months (95% CI 18.3–26.2) in patients who received subsequent therapy (*p* < 0.001) (Figure 2). Baseline ECOG performance status was the only characteristic found to be associated with improved OS in multivariate analysis (Table 3). Median OS was 19.6 months (95% 16.0 to 23.1) in patients with baseline ECOG performance status 0–1, while median OS was 6.1 months (95% 4.7 to 7.6) in patients with performance status ≥ 2. Survival was calculated for patients who had progression on first-line pembrolizumab from the date of the last dose of pembrolizumab to understand the impact of second-line therapy. The median OS for BSC was 2.1 months versus 13.3 months for second-line or greater treatment (*p* < 0.001) (Figure 3).

## 4. Discussion

In our real-world advanced NSCLC PD-L1 ≥ 50% population treated with first line pembrolizumab, only 21% of patients with disease progression received second-line systemic therapy. Predictors of subsequent treatment include patients diagnosed at a younger age and with good baseline ECOG performance status. The median OS was significantly improved in patients who were able to receive second-line treatment compared to patients who received the best supportive care. The difference in survival outcomes may reflect underlying cancer biology, patients’ baseline status, response to first line treatment, and the importance of subsequent lines of therapy.

The introduction of immunotherapy into multiple malignancy types came with enthusiasm, especially when a subset of patients were found to have meaningful long-term survival. In NSCLC, immunotherapy was rapidly adopted and has been found to improve 1- and 5-year lung cancer-specific survival while also improving quality of life. However, despite all patients having PD-L1 ≥ 50%, currently the best biomarker for selection, in our real-world cohort, 27% of patients discontinued pembrolizumab at the 9-week mark. In the KEYNOTE-024 study, in both the pembrolizumab and platinum chemotherapy arms, the progression rate at the first evaluation scan was similarly just under 30%, suggesting that regardless of first-line therapy, a proportion of patients are destined to have poor outcomes. Different from KEYNOTE 024, however, our population included 39% of ECOG performance status ≥ 2 patients, which likely further deteriorated with cancer progression, limiting the ability to deliver subsequent therapies. With the introduction of lung cancer screening and navigator pathways, it is anticipated that patients will be referred earlier in their disease trajectory, enabling more treatment options and treatment initiation when patients have a better performance status.

Hyperprogressive disease is generally defined as rapid cancer growth during a treatment period with appropriately selected treatment. Unfortunately, a subset of patients who receive first-line pembrolizumab will experience hyperprogressive disease, which has been reported in as many as 37% of lung cancer patients [13]. When considering immunotherapy as an alternative to chemotherapy, the rapidly progressive disease has also been documented at a much higher rate in those who receive immunotherapy [13]. Currently, a valid clinical or molecular marker has not been identified to pre-select patients who will experience significant early progression of the disease, a poor prognostic marker. Close monitoring for clinical worsening and early imaging are key to identifying patients who will not benefit from immunotherapy and ensuring a rapid treatment switch.

Attrition of second-line systemic therapy after progression on first-line agents is seen in all malignancy types; however, it is especially evident in lung cancer [3]. In this real-world evaluation, just 21% of patients with documented disease progression received second-line systemic therapy, with most patients being treated with platinum-based chemotherapy, which is lower than expected. In KEYNOTE-024, 53% of patients received second-line therapy, with most patients receiving chemotherapy [8]. Velcheti et al. examined the real-world uptake of second-line therapy after first-line pembrolizumab and found 32% received therapy, 35% received third-line therapy, and 25% received fourth-line therapy [14]. Cortellini et al. specifically evaluated post-pembrolizumab monotherapy treatment in a stage IV NSCLC population with PD-L1 ≥ 50%, and 44% received further systemic therapy. In their study, patients were less likely to receive any subsequent therapy if they were older, had worse ECOG performance status, or required steroids prior to starting pembrolizumab [15]. In our cohort, patients diagnosed at a younger age and with good baseline ECOG performance status were more likely to receive second-line systemic therapy, while sex, smoking status, and histology did not impact second-line delivery. It is notable that in prior studies in the British Columbia population, 55% of patients received second line chemotherapy when first-line platinum doublet was the standard of care [16]. The low rate of second-line systemic therapy uptake after first-line immunotherapy is multifactorial, as patients may have had further deterioration in performance status, patients may have had preferences to avoid chemotherapy, and care providers may have had toxicity concerns.

Attrition in lung cancer also varies depending on the available systemic therapy selection. When less toxic systemic therapy options are present, the uptake of second-line therapy may be higher. Specifically, when the second-line treatment option was only docetaxel, the uptake was just 21%; however, when pemetrexed became available, the second-line treatment uptake increased to 55% [16]. In the scenario of first-line ICI, platinum doublet chemotherapy as second-line may be a more challenging transition for patients. Uptake of second-line therapy is higher in NSCLC patients with driver mutations. Park et al. considered a pooled population from LUX-Lung 3, 6, and 7. Here, they found that 71% of patients with EGFR mutant NSCLC with progression on afatinib received second-line therapy, with the majority of patients receiving platinum doublet chemotherapy [17]. It is unclear why this difference exists, although EGFR mutations are associated with never-smoking status, and these patients may have had fewer comorbidities that would impact the delivery of a second-line platinum doublet.

In this evaluation, patients who received first-line pembrolizumab had a median OS of 14.0 months from initiation of systemic therapy, which is substantially shorter than the median OS found in KEYNOTE-024 of 26.3 months [7,8]. Other retrospective real-world evaluations have similarly found shorter OS compared to clinical trial outcomes, with cohorts from the United States of America and Europe having a median OS ranging from 15.8 to 22.0 months [14,15,18,19,20]. It is sobering that the real-world OS benefits of first line immunotherapy are significantly different from those of the trial, although this is explained in part by the selection criteria regarding performance status, age, and comorbidities in addition to subsequent treatments. In our study, the median OS for patients who received subsequent therapies was 22.2 months versus 5.6 months with the best supportive care. There are confounding factors for the significant numerical difference in OS; in addition to better baseline status and younger age, patients who are able to receive subsequent therapies are also more likely to have benefited from their first-line treatment. Patient and physician expectations with respect to the translation of clinical trial improvements into clinical practice need to be tempered.

Extrapolating clinical trial results, with strict inclusion and exclusion criteria, into clinical practice is a difficult process. Frequently, clinical trials only include patients without comorbidity and with ECOG performance status 0–1, but in clinical practice, we commonly extend these results to patients with performance status 2 without truly understanding if there is benefit in less functional patients. This is especially important in NSCLC, as 30–40% of the population has ECOG performance status 2 [21]. IPSOS was a phase III study comparing first-line atezolizumab to single-agent vinorelbine or gemcitabine for patients with ECOG PS of 2 or 3, or age ≥ 70 years with significant comorbidities or other contraindications to platinum chemotherapy regardless of PD-L1 status [22]. The median OS was 10.3 months with atezolizumab versus 9.2 months with single-agent chemotherapy, and PDL1 levels did not appear to affect the outcomes. Serious treatment-related adverse events occurred in 11.7% versus 15.6% of patients, respectively, with similar drug discontinuation rates in both arms. The IPSOS study provides guidance for expected outcomes in patients with poor functional status; however, a prospective trial including patients with ECOG performance status 0–1 and 2 is required to conclusively understand the efficacy and toxicity differences. In our study, when considering OS in patients with performance status 0–1 compared to ≥2, patients with poor performance status had a significantly shorter OS with a median of just 19.6 months compared to 6.1 months. However, our results are not alone; multiple other groups have considered response to anti-PD-1/PD-L1 retrospectively in NSCLC with ECOG performance status 2 and similarly found disappointing response rates, progression-free survival, and OS [21,23]. Ultimately, it is not known to what extent PDL1 ≥ 50% of patients with performance status 2 benefits from single-agent pembrolizumab. Patients with a more limited functional status represent a heterogeneous group, and further classification of these patients may be needed.

Limitations of this study include the retrospective nature of the study design. Patients were only included if they had been referred to BC Cancer, so this is not capturing all stage IV NSCLC treated with first-line pembrolizumab within British Columbia. ECOG performance status and evidence of disease progression were collected through manual chart review, which is limited by physician documentation accuracy. PD-L1 status was not evaluated through a centralized evaluation, therefore lacking standardization. There was a lack of information on the use of other medications, such as steroids, that could influence immunotherapy outcomes. Other prognostic factors that may impact the receipt of second-line systemic therapy, including comorbidity or laboratory values, were not collected. The primary strength of this study is that it reflects the real-world experiences of a stage IV NSCLC population with high PD-L1 expression.

## 5. Conclusions

In our stage IV NSCLC population, we found that OS was shorter than identified in KEYNOTE-024, but our results were similar to other real-world datasets. Within our population, only 21% of patients received second-line therapy; however, those who received subsequent lines of systemic therapy had a mean survival of 22.2 months. Although differences exist between a clinical and non-clinical trial population, important considerations for future improvements include early referral of patients who present with a better performance status, discussion about the potential for future chemotherapy with patients, and timely transition to second-line therapy at progression.

## Figures and Tables

**Table 1:** Baseline characteristics of Stage IV NSCLC with PDL1 ≥ 50% whole cohort, patients with disease progression, disease progression with second line treatment, disease progression followed by best supportive care.

*N* (%)	Overall (*n* = 718)	Patients with Progression (*n* = 567)	Progression Followed by Second Line Therapy (*n* = 118)	Progression Followed by BSC (*n* = 449)	*p*-Value for Second Line Treatment Versus BSC
Age (median), years	70	70	67	71	<0.001
Sex					
Male	331 (46%)	260 (46%)	51 (43%)	209 (47%)	
Female	387 (54%)	307 (54%)	67 (57%)	240 (53%)	0.519
Histology					
Non-Squamous	573 (80%)	437 (77%)	97 (82%)	340 (76%)	
Squamous	145 (20%)	130 (23%)	21 (18%)	109 (24%)	0.136
Smoking Status					
Never	44 (6%)	40 (7%)	12 (10%)	28 (6%)	
Former	521 (73%)	410 (72%)	82 (70%)	328 (73%)	
Active	132 (18%)	103 (18%)	20 (17%)	83 (19%)	
Unknown	21 (3%)	14 (3%)	4 (3%)	10 (2%)	0.416
Smoking years (median)	40	40	35	40	0.217
Baseline ECOG PS					
0–1	436 (61%)				
≥2	280 (39%)	324 (57%)	90 (76%)	234 (52%)	
Unknown	2 (<1%)	243 (43%)	28 (24%)	215 (48%)	<0.001
CNS Metastases	65 (9%)	56 (10%)	10 (9%)	46 (10%)	0.566
Mutation (tested *n* = 600)					
KRAS	110 (32%)	80 (30%)	18 (31%)	62 (30%)	
BRAF	15 (4%)	12 (5%)	1 (2%)	11 (5%)	
KRAS and BRAF	4 (1%)	2 (1%)	0 (0%)	2 (1%)	
Other	8 (2%)	6 (2%)	2 (3%)	4 (2%)	0.672
Median duration pembrolizumab treatment, months (IQR)	4.44 (1.38–12.92)	2.99 (0.69–7.23)	5.37 (2.76–11.59)	2.63 (0.69–6.31)	<0.001

**Table 2:** Multivariable analysis for characteristics associated with receipt of second line systemic therapy.

	OR (95% CI) Multivariate Analysis	*p*-Value
Age	0.949 (0.927–0.972)	<0.001
ECOG PS 0–1 versus ≥ 2	2.591 (1.610–4.167)	<0.001
Duration of Pembrolizumab	1.053 (1.023–1.085)	<0.001

**Table 3:** Univariate and multivariate analysis for OS.

	HR (95% CI) Univariate Analysis	*p*-Value	HR (95% CI) Multivariate Analysis	*p*-Value
Age	1.001 (0.991–1.011)	0.872	0.997 (0.987–1.007)	0.520
Sex				
Female versus Male	1.071 (0.888–1.291)	0.473	1.041 (0.863–1.256)	0.675
ECOG PS				
0–1 versus ≥ 2	1.894 (1.567–2.229)	<0.001	1.892 (1.562–2.291)	<0.001
0–1 versus unknown	1.336 (0.728–2.45)	0.349	1.459 (0.790–2.695)	0.227
Pembrolizumab schedule				
Q3W versus Q6W	0.616 (0.368–1.032)	0.066	0.637 (0.378–1.072)	0.089

## Data Availability

Data will be made available upon reasonable request and ethics review.
